# The vaccination coverage rate in under-5 children in Nasiriyah, Iraq before and during the COVID-19 pandemic

**DOI:** 10.4178/epih.e2022035

**Published:** 2022-03-14

**Authors:** Ali Rifaat Alhaddad, Elham Ahmadnezhad, Akbar Fotouhi

**Affiliations:** 1 Department of Epidemiology and Biostatistics, School of Public Health, Tehran University of Medical Sciences, Tehran, Iran; 2 National Institute of Health Research (NIHR), Tehran University of Medical Sciences, Tehran, Iran

**Keywords:** Vaccination, COVID-19, Coverage rate, Primary healthcare, Pandemics, Universal health coverage

## Abstract

**OBJECTIVES:**

This study compared the vaccination coverage rate (VCR) in children under 5 years old in Nasiriyah, Iraq before and during the coronavirus disease 2019 (COVID-19) pandemic.

**METHODS:**

This cross-sectional study was conducted in the city of Nasiriyah in southeastern Iraq, with data collected from 79 primary healthcare facilities. This study evaluated the VCR in 3 periods (2018, 2019, and 2020) using multi-level random sampling. Pertinent data were extracted from the vaccination records of 598 children for Bacillus Calmette-Guérin (BCG); pentavalent 1, 2, and 3; measles; and activated oral poliovirus vaccine 1 and 2. Missing data were completed by telephone calls to participants’ parents. Logistic regression was applied to compare and estimate the odds ratios (ORs) with 95% confidence intervals (CIs) for the association between VCR and related factors.

**RESULTS:**

The data showed the greatest decline in the studied vaccines in 2020. Among the vaccines studied, BCG had the highest rate in all 3 periods (100% VCR) and measles had the lowest rate (83.7%), reaching 63.6% in 2020 (p<0.001). The highest OR among all types of vaccine were found for the pentavalent-3 vaccine among city dwellers and those born in 2020 (OR, 2.67; 95% CI, 1.39 to 5.10 and OR, 2.34; 95% CI, 1.28 to 4.28, respectively).

**CONCLUSIONS:**

The VCR for children decreased during the COVID-19 pandemic in Iraq, and new health policies are needed to increase the coverage rate. Improving the knowledge and attitudes of parents, as well as removing barriers or risk factors, can also be effective in improving the VCR.

## INTRODUCTION

In December 2019, the emergence of the new severe acute respiratory syndrome coronavirus 2 was first reported in Wuhan, China [1], and it spread quickly to the entire world in a short amount of time. The World Health Organization (WHO) declared a state of emergency that aroused international attention on January 30, 2020, and declared the coronavirus disease 2019 (COVID-19) a global pandemic on March 11, 2020 [2].

Individual, demographic, and political factors such as lockdowns, transportation restrictions, health facility and health staff capacities, postponement of patient treatments due to concerns about exposure to COVID-19, or a combination of these factors and other factors affected healthcare services [3-5]. The challenge has been even more complex in Iraq. Political unrest and regime change, the actions of the Islamic State of Iraq and Syria, war, sanctions, and internal conflicts have affected the health system and complicated the pandemic.

Vaccination programs are among the essential services provided at health centers. Regarding continuity of health coverage, the WHO reported that approximately 8 months into the COVID-19 pandemic, 90% of countries reported disruption in some essential healthcare services including vaccinations, especially in the earlier phases of COVID-19 [6]. In Iraq, essential healthcare services (including children’s healthcare) are the responsibility of public primary healthcare centers, which cover the entire population free of charge. All children covered by public centers can receive health services (including vaccinations); therefore, utilization of healthcare services is expected to be high [7].

Vaccines are considered one of the most important successes in the history of public health and prevention of vaccine-preventable diseases [8]. Extensive vaccine coverage in the world, especially in developing countries, has contributed to increased child survival rates, reduced rates of infection, and reduced long-term disability; therefore, any declines in vaccination rates pose tremendous public health risks to children [9,10]. As one of the countries affected by the pandemic, periodic peaks in COVID-19 cases have made Iraq one of the top 20 countries in the world in terms of infection rates, with more than 2 million people affected and approximately 24,000 deaths as of February, 2022 [11]. Iraq ranks 118th in deaths per million people among all countries. In response to the pandemic, the National Crisis Committee was formed by the Ministry of Health and this committee was then established in all provinces. Travel restrictions were imposed, air and land borders were closed, and passenger entry was controlled [12].

Since the Iraqi health system applied all its resources to respond to this situation, disruptions of other services, especially ambulatory services, were likely. In addition, people were hesitant to go to health facilities for fear of getting infected. Although children’s’ routine immunizations are an essential health service and should not be delayed, conditions during the pandemic have affected this service. Studies in other settings have reported this issue as well [13-16].

Evaluation of the vaccination coverage in children is important information, necessary for policy makers to define the problem and plan solutions. This study was conducted to evaluate and compare the vaccination coverage rate (VCR) in children in Nasiriyah, Iraq before and during the COVID-19 pandemic.

## MATERIALS AND METHODS

### Study area

This retrospective study was conducted in Nasiriyah, Iraq. The population of Nasiriyah is 634,500, and approximately 400,000 inhabitants live in urban areas with the rest in rural areas. Approximately 20% of the residents in Nasiriyah were under 5 years old [17]. The Nasiriyah health department includes 9 primary healthcare districts and 79 healthcare facilities. In this study, we randomly selected 3 health districts (Nasiriyah 1, Nasiriyah 2, and Gharaff) and 3 healthcare centers in each health district (a total of 9 healthcare facilities were included).

### Participants and data sources

The inclusion criteria for this study were children under 5 years of age, residing in Nasiriyah, and having a vaccination document in 2018 and 2019 (baseline or pre-pandemic period), and 2020 (later pandemic period). Exclusion criteria were a missing address and telephone number, missing significant vaccine data in the document, and participants who did not provide informed written consent.

Proportion-to-size sampling was conducted in each health district. A list of clients from these 9 healthcare centers was collected and participants were randomly selected (simple random sampling). The overall sampling was multi-stage random sampling.

All information came from electronic health records. After selecting the participants, we contacted their parent(s) and, after describing the objectives of the research and ensuring individual privacy, informed consent was obtained from the parents. Missing data in the document was completed by calling the participants’ parent(s) and obtaining the data from the vaccine card. A standard questionnaire was used to collect information. This questionnaire is part of the Multiple Indicator Cluster Survey questionnaire used in 2018 in Iraq [18]. The vaccine types, dates of injection, participants’ age and sex, and mothers’ education level were collected and recorded on a data form created by the researcher. The evaluated vaccines were Bacillus Calmette–Guérin (BCG), pentavalent-1, pentavalent-2, pentavalent-3, measles, activated oral polio vaccination (OPV)-1, and activated OPV-2.

The criteria for vaccine completeness in this study was that the child was vaccinated at any time prior to the survey, according to vaccine documents or parents’ reports.

The relatively large sample size, multi-center coverage in the rural and urban districts of Nasiriyah city, double-checking of the data, and quality control of the data by members of the research project, contribute to the authors’ high confidence in the validity and reliability of their findings in this targeted population.

### Statistical analysis

The mean and standard deviation (SD) were used to describe quantitative variables. Frequency and percentage were used to describe qualitative variables. Analysis of variance was used to compare the proportion of vaccine injections in the 3 periods (2018, 2019, and 2020).

The VCR was calculated as the number of children who had received the recommended doses of the specific vaccine for their age milestone, divided by the number of children who were eligible to receive the vaccine up to 36 months old.

Logistic regression was used to calculate the odds ratio (OR) of vaccination as a measure of association, together with 95% confidence intervals (CIs). Two-tailed analyses were conducted, and a p-value less than 0.05 was considered to indicate statistical significance.

### Ethics statement

Approval for the study was obtained from the Ethics Committee of the Tehran University of Medical Sciences (IR.TUMS.SPH.REC.1400.077). Official approvals were obtained from the Central Health Department in Nasiriyah. In addition, informed consent was obtained from all participants. No additional cost or harm was imposed on participants and, to protect privacy, no personal data such as phone numbers or personal names were recorded.

## RESULTS

This study included 598 children under the age of 5 years, of whom 49.7% (n=297) were boys and 50.3% (n=301) were girls. The mean age of the participants was 19.31±8.46 months (95% CI, 18.63 to 19.99). The majority of study subjects lived in urban areas (70.7%; n=424), and the rest of the data was extracted from the Gharaaf healthcare center (a rural area). Most participants (81%; n=489) were born in hospitals with the rest born in other locations, assisted by local midwives. Additional information on the participants’ and their families’ baseline characteristics in the 3 districts is shown in Table 1.

Table 2 presents the VCR of each vaccine in the 3 periods. The coverage rates of BCG were the same in 2018, 2019, and 2020 and all participants born in each year received their vaccines at the hospital. Some participants received the BCG vaccine 1 week after birth, especially children born outside of the hospital. In addition, the trend of the VCRs for pentavalent-1 (2018: 96.6%, 2019: 95.3%, 2020: 88.6%; p=0.005), pentavalent-2 (2018: 91.0%, 2019: 88.8%, 2020: 75.0%; p<0.001), and pentavalent-3 (2018: 93.1%, 2019: 87.6%, 2020: 69.7%; p<0.001) showed statistically significant decreases. The VCRs for measles also showed statistically significant decreases (2018: 83.7%, 2019: 69.1%, 2020: 63.6%; p<0.001). Finally, statistically significant decreases were also observed in the VCRs for activated OPV-1 and measles that were administered at age 12 months (2018: 97.4%, 2019: 91.0%; p<0.001) (Table 2).

The univariable logistic regression analysis of potential predictors of receiving the vaccines is shown in Table 3. The OR of receiving pentavalent-1 among female children was 0.34 compared to male children (OR, 0.34; 95% CI, 0.16 to 0.75; p=0.007). The OR of receiving pentavalent-2 in participants that received pentavalent-1 was 12 relative to participants who had not received pentavalent-1 (OR, 12.00; 95% CI, 5.76 to 24.99; p<0.001).

In addition, the OR of receiving pentavalent-3 for participants in rural areas was 2.34 compared to participants in urban areas. This difference was statistically significant (OR, 2.34; 95% CI, 1.28 to 4.28; p=0.006). The date of vaccination also had an association with receiving pentavalent-3. The OR of receiving a vaccine in 2019 was 0.52 compared to 2018 (OR, 0.52; 95% CI, 0.27 to 0.98; p=0.044) and the OR of receiving pentavalent-3 in 2020 was 0.17 times when using 2018 as the reference data (OR, 0.17; 95% CI, 0.09 to 0.32; p<0.001). The OR of receiving pentavalent-3 in participants who received the previous vaccines was 10.51 times compared to participants who had not received pentavalent-2 (OR, 10.51; 95% CI, 6.16 to 17.94; p<0.001). Finally, the OR of receiving the measles vaccine in girls was 1.49 compared to boys (OR, 1.49; 95% CI, 1.03 to 2.15; p=0.033).

Regression analysis based on the mothers’ education level did not yield statistically significant results.

## DISCUSSION

This study was conducted to compare the VCR in the children of Nasiriyah, Iraq before and during the COVID-19 pandemic. To the best of our knowledge, this study was the first to evaluate the effect of the COVID-19 pandemic on the routine VCR of children in Iraq.

Our study showed a dramatic decrease in all VCRs during the COVID-19 pandemic except BCG. In 2019, the rate of decline was higher than in 2018 and the 2020.

The results of this study concur with other studies worldwide, showing that a decline in routine pediatric vaccination rates has occurred since the beginning of the pandemic. A study in Lebanon [19] reported that the COVID-19 pandemic reduced the VCR for routine childhood vaccinations reduced by 31%. The measles-mumps-rubella (MMR) vaccine coverage rate in England declined by nearly 20% [20]. A study in the United States found that VCRs decreased in all age cohorts, except for the hepatitis B vaccination that is administered at birth in the hospital setting [21]. Rana et al. [22] reported a 25% decrease in the VCR in Pakistan.

This is an important finding, and although the reduction varies by vaccine type, it should be addressed promptly by local and international health authorities. An important risk-benefit analysis showed that the prevented deaths among children due to routine childhood immunizations is higher than the COVID-19 related deaths [11]. Therefore, health facilities need to intervene and prioritize vaccination coverage in all areas and ages.

The measles VCR decreased proportionately more than other vaccines in this study. The measles vaccine is given in the form of the MMR vaccine at 1 year of age. The rate of this vaccine in the baseline period was also lower than other vaccines. This presents the possibility of measles epidemics, which could further complicate the pandemic. In April 2020, the United Nations Children’s Fund (UNICEF) warned that routine vaccinations in Iraq had been missed [23]. The UNICEF report also revealed that the measles vaccine coverage could reduce by an additional 20% in Iraq during the pandemic. To deal with this situation, in 2020, UNICEF and the WHO called on the government of Iraq to increase its investment in health services and to start planning intensified immunization campaigns to identify and reach children with missed vaccine doses once the pandemic is fully under control. Although our study showed that in 2019 the measles vaccine rate dropped approximately 24%, the situation has improved somewhat in 2020. This issue needs serious attention, and children who have not yet received the measles vaccine should be identified and vaccinated at the earliest opportunity.

Our results showed a significant association between children who were vaccinated during the COVID-19 pandemic and a history of delayed vaccinations during and before the COVID-19 pandemic. Other studies found that parents who made receiving vaccinations a high priority for their children before the COVID-19 pandemic, had the same level of commitment in the COVID-19 pandemic period [24,25].

There are various reasons why COVID-19 has affected the VCR. For example, regional variations in VCR reduction have also been shown in England and Pakistan [20,22] and this diversity may be due to cultural differences in the extent to which health protocols are followed, infection concerns, and socioeconomic status differences. In addition, previous studies have shown that rural residence is associated with low socioeconomic indicators (e.g., poverty and low education level) [26,27].

Other possible factors in the VCR decline were COVID-19 restrictions that forced people to stay at home [28], in addition to parental concerns about potentially exposing their children to infection during health center visits [29,30]. Health policy decisions such as isolation and quarantine during the pandemic and changes in public health priorities have also contributed to reducing the VCR. The factors associated with delays in the vaccination process likely include political decisions, fear of being exposed to the COVID-19 virus in health centers [31], socioeconomic status [32], and concerns about the safety of vaccines and their potential side effects [33].

Improved vaccination rates in primary care are feasible when parents feel safe and are kept informed of the timing of vaccinations. Healthcare workers also require access to systems for contacting and following up with families [34].

Our study had several limitations. The study was conducted in a selected area in Iraq and may not represent the total Nasiriyah population. Due to insufficient data availability, measurement inequities were not evaluated. The results of this study provide a snapshot of the VCR during the pandemic in Iraq and did not evaluate the most current VCR. Although coverage of the population in the studied health facilities was adequate, there may be children who were not covered by the centers and this group may have had the highest drop in vaccination rate. A population-based study will help to make more accurate estimates. Our study did not assess the hepatitis B and influenza vaccines, which we recommend addressing in future studies.

In conclusion, the routine VCR for under-5 children in Nasiriyah, Iraq decreased during the pandemic. A house-to-house evaluation, vaccination campaigns, and a proactive vaccine surveillance system are recommended to achieve a near-100% rate for all vaccines in Iraq, based on the results of this study. The lost vaccinations may have long-term consequences, making it important to engage families in taking advantage of every opportunity to catch up on missed vaccines. Meanwhile, launching public vaccine mobilization campaigns at short intervals is a solution that the Iraqi government might consider. Although some factors affecting the VCR (e.g., baseline factors such as maternal literacy) were assessed in this paper, we would suggest further study to evaluate other possible factors such as socioeconomic status and birth rank.

The VCR has fallen during the COVID-19 pandemic in Nasiriyah; therefore, the healthcare system must take immediate action and provide active vaccination programs to prevent dangerous outcomes.

## Figures and Tables

**Table 1.** Baseline characteristics of the participants and families in 3 sub-districts

Characteristics	Nasiriyah 1 (n=206)	Nasiriyah 2 (n=216)	Gharaaf (n=176)	Total (n=598)	p-value
Age, mean±SD (mo)	19.12±8.25	18.33±8.07	20.71±8.99	19.31±8.46	0.019
Sex					0.332
Male	106 (51.7)	108 (50.2)	83 (46.6)	297 (49.7)	
Female	99 (48.3)	107 (49.8)	95 (53.4)	301 (50.3)	
Mother’s education level					<0.001
Uneducated	0 (0.0)	0 (0.0)	11 (6.2)	11 (1.8)	
1-8 yr of education	45 (22.0)	41 (19.1)	62 (34.8)	148 (24.7)	
Diploma	136 (66.3)	129 (60.0)	96 (53.9)	361 (60.4)	
Bachelor’s degree	16 (7.8)	28 (13.0)	9 (5.1)	53 (8.9)	
Master of sciences and higher	8 (3.9)	17 (7.9)	0 (0.0)	25 (4.2)	

SD, standard deviation.

**Table 2.** The vaccination coverage rate of each vaccine in 2018, 2019, and 2020

Variables	Vaccination coverage rate				p-value (trend in 2018, 2019, and 2020
	2018 (n=233)	2019 (n=233)	2020 (n=132)	Total (n=598)	
BCG	233 (100)	223 (100)	132 (100)	598 (100)	0.999
Pentavelent-1	225 (96.6)	222 (95.3)	117 (88.6)	564 (94.3)	0.005
Pentavelent-2	212 (91.0)	207 (88.8)	99 (75.0)	518 (86.6)	<0.001
Pentavelent-3	217 (93.1)	204 (87.6)	132 (69.7)	553 (92.4)	<0.001
Measles	195 (83.7)	161 (69.1)	84 (63.6)	440 (73.6)	<0.001
Activated OPV-1	227 (97.4)	212 (91.0)	NA^1^	439 (73.4)	<0.001
Activated OPV-2	212 (91.0)	NA^1^	NA^1^	212 (91.0)	<0.001

BCG, Bacillus Calmette-Guérin; OPV, oral polio vaccine.

1 Children who were born in 2019 and 2020 had not received vaccines at the time of data-gathering.

**Table 3.** Associations between receiving a vaccine and potential predictors

Variables			OR (95% CI)	SE	p-value	Variables			OR (95% CI)	SE	p-value
Pentavalent-1						Pentavalent-3					
	Sex			0.40	0.007		Sex			0.23	0.960
		Male	1.00 (reference)					Males	1.00 (reference)		
		Female	0.34 (0.16, 0.75)					Female	0.99 (0.62, 1.56)		
	Residence			0.38	0.998		Residence			0.31	0.006
		Urban	1.00 (reference)					Urban	1.00 (reference)		
		Rural	0.99 (0.47, 2.13)					Rural	2.34 (1.28, 4.28)		
	Birth year						Birth year				
		2018	1.00 (reference)					2018	1.00 (reference)		
		2019	0.61 (0.19, 1.93)	0.59	0.397			2019	1.25 (0.75, 2.09)	0.26	0.384
		2020	1.09 (0.36, 3.34)	0.57	0.875			2020	2.67 (1.39, 5.10)	0.33	0.003
	Mother’s education level						Mother’s education level				
		Uneducated and primary school	1.00 (reference)					Uneducated and primary school	1.00 (reference)		
		High school and diploma	1.80 (0.85, 3.80)	0.38	0.123			High school and diploma	0.71 (0.40, 1.25)	0.29	0.710
		Higher than diploma	1.65 (0.52, 5.23)	0.59	0.397			Higher than diploma	0.70 (0.32, 1.54)	0.40	0.702
Pentavalent-2						Measles					
	Sex			0.24	0.949		Sex			0.19	0.033
		Male	1.00 (reference)					Male	1.00 (reference)		
		Female	1.02 (0.63, 1.63)					Female	1.49 (1.03, 2.15)		
	Residence			0.26	0.905		Residence			0.11	0.128
		Urban	1.00 (reference)					Urban	1.00 (reference)		
		Rural	0.97 (0.58, 1.62)					Rural	0.74 (0.50, 1.09)		
	Birth year						Birth year				
		2018	1.00 (reference)					2018	1.00 (reference)		
		2019	0.91 (0.39, 2.10)	0.43	0.824			2019	0.77 (0.50, 1.19)	0.22	0.240
		2020	0.79 (0.37, 1.69)	0.38	0.556			2020	0.94 (0.50, 1.78)	0.32	0.853
	Mother’s education level						Mother’s education level				
		Uneducated and primary school	1.00 (reference)					Uneducated and primary school	1.00 (reference)		
		High school and diploma	0.87 (0.50, 1.52)	0.28	0.636			High school and diploma	0.77 (0.50, 1.19)	0.22	0.237
		Higher than diploma	1.10 (0.48, 2.55)	0.43	0.818			Higher than diploma	0.94 (0.50, 1.78)	0.03	0.853

OR, odds ratio; CI, confidence interval; SE, standard error.
